# Responsibility Without Freedom? Folk Judgements About Deliberate Actions

**DOI:** 10.3389/fpsyg.2019.01133

**Published:** 2019-05-21

**Authors:** Tillmann Vierkant, Robert Deutschländer, Walter Sinnott-Armstrong, John-Dylan Haynes

**Affiliations:** ^1^Bernstein Center for Computational Neuroscience, Charité – Universitätsmedizin Berlin, Corporate Member of Freie Universität Berlin and Humboldt-Universität zu Berlin, Berlin, Germany; ^2^School of Philosophy, Psychology and Language Sciences, University of Edinburgh, Edinburgh, United Kingdom; ^3^Department of Philosophy, Kenan Institute for Ethics, Duke University, Durham, NC, United States; ^4^Berlin Center for Advanced Neuroimaging & Department of Psychology & Berlin School of Mind and Brain, Humboldt-Universität zu Berlin, Berlin, Germany

**Keywords:** freedom, responsibility, deliberation, consequence, experimental philosophy

## Abstract

A long-standing position in philosophy, law, and theology is that a person can be held morally responsible for an action only if they had the freedom to choose and to act otherwise. Thus, many philosophers consider freedom to be a necessary condition for moral responsibility. However, empirical findings suggest that this assumption might not be in line with common sense thinking. For example, in a recent study we used surveys to show that – counter to positions held by many philosophers – lay people consider actions to be free when they are spontaneous rather than being based on reasons. In contrast, responsibility is often considered to require that someone has thought about the alternative options. In this study we used an online survey to directly test the degree to which lay judgements of freedom and responsibility match. Specifically, we tested whether manipulations of deliberation affect freedom and responsibility judgements in the same way. Furthermore, we also tested the dependency of these judgements on a person’s belief that their decision had consequences for their personal life. We found that deliberation had an opposite effect on freedom and responsibility judgements. People were considered more free when they acted spontaneously, whereas they were considered more responsible when they deliberated about their actions. These results seem to suggest that deliberating about reasons is crucially important for the lay concept of responsibility, while for the lay notion of freedom it is perceived to be detrimental. One way of interpreting our findings for the interdisciplinary debate on free will and responsibility could be to suggest that lay beliefs match the philosophical position of semi-compatibilism. Semi-compatibilists insist that the metaphysical debate on the nature of free will can be separated from the debate on conditions of responsible agency. According to our findings the beliefs of lay people are in line with views held by semi-compatibilists, even though we did not test whether they endorse that position explicitly.

## Introduction

Philosophers have been analyzing the relation of free will and responsibility since antiquity. Most of them have proposed that freedom is a necessary condition for responsibility (Van Inwagen, 1983; Kant, 1998; Aristotle, 2000; Augustine, 2006; Vihvelin, 2008). Many philosophers furthermore claim that people act freely or autonomously only if they act for reasons (Locke, 1975; Kant, 1998), or only if they are provided with options with different values (Van Inwagen, 1989; Kane, 2005; Schlosser, 2014; Mecacci and Haselager, 2015), or only if the action has significant consequences for their personal life (Roskies, 2011; Schlosser, 2014; Mecacci and Haselager, 2015).

Recent empirical research has shown that lay people’s beliefs do not agree with these conceptual positions. In one study (Deutschländer et al., 2017) we found that the deliberation of reasons, the availability of different choice options, or the existence of real life consequences were all not necessary for an action to be considered free. On the contrary, lay people judged actions to be most free if (a) they were chosen without deliberation, (b) they involved different (as opposed to equal) options, and (c) they were believed to have different real-life consequences. Thus, paradoxically, deliberation was even considered to reduce freedom, counter to the notion that reasons play a key role in assigning freedom to actions.

Please note that this research pertained to subjective ratings of freedom rather than responsibility. For lay concepts of responsibility, in contrast, deliberation might non-etheless be important, but this hasn’t been empirically tested so far. Previous studies have already jointly measured the effects of experimental conditions on free will and moral responsibility judgements (Nahmias et al., 2005, 2007), however, regarding somewhat different experimental manipulations than here (see section “Discussion”).

Thus, here, we directly compare how freedom judgements and responsibility judgements of laypeople are affected by the factors deliberation, choice, and consequence. We compare how the following factors affect judgements of freedom versus responsibility: (1) Whether an action was spontaneous or based on deliberation; (2) whether the decision involved qualitatively different options (choosing) or identical options (picking) [for the distinction between picking and choosing see Ullmann-Margalit and Morgenbesser (1977)]; (3) whether the subsequent actions led to consequences for a person’s life or not. As Nahmias et al. (2005) note, it is very difficult to ask subjects about abstract theories like determinism and compatibilism that are quite far removed from everyday life without biasing their answer in crafting the vignettes. Instead, we have opted to test action types that are both clearly relevant for the free will discussion, but also easily understandable in an everyday context.

## Materials and Methods

### Participants

We deployed an online-questionnaire via university email distribution-lists. We received responses of 133 participants (62.6% female, 31.3% male, 3.8% missing values). The age of the respondents ranged from 18 to 53 years (*M*_age_ = 25.03 years, *SD*_age_ = 7.76 years). Almost all respondents (97.7%) had a high-school or university degree. 66.7% of the respondents had not previously thought about the question of free will, while the remaining third had (“Have you ever thought about free action or free will?”). The research was approved by the psychological ethics committee of the Humboldt University in accordance with the declaration of Helsinki. Informed consent was obtained at the beginning of the online-questionnaire.

### Material and Procedure

We asked participants to respond to questions in an online questionnaire containing short written scenarios. We implemented those scenarios in the software Unipark (Questback GmbH, Köln, Germany). Each questionnaire contained eight scenarios. Those scenarios followed from a combination of three within-subject factors: deliberation, choice, and consequence. The factor *deliberation* used two levels: A person either deliberated about their choice or acted spontaneously. The factor *choice* included two levels: “choosing” among different options or “picking” among identical options. The factor *consequence* had two levels: Participants knew that the action either had significant consequences for a person’s life (signing a job contract) or it involved an insignificant action with no consequences (taking a note) (see Table 1 for all scenarios used in this study based on all possible combinations of the three factors). Before starting the questionnaire participants were randomly assigned to one of two possible groups: One group was asked to provide only freedom ratings, the other group was required to provide only responsibility ratings. This between-subjects approach in our mixed design was adopted in order to avoid priming the participants to the purpose of the study.

**Table 1:** The three within-subject factors and the corresponding scenarios.

Within-subject factors			Operationalization
Deliberation	Choice	Consequence	Scenario
Deliberation	Choosing	Yes	Matthias looks for a new long-term job. He has gotten two job offers. For both jobs he received contracts for signing. The job conditions are very different. Matthias deliberates what job would be better. Only after careful pondering he decides for a job and signs one contract.
Deliberation	Choosing	No	Matthias looks for a pen to take a rather unimportant note. On the desk in front of him he sees two very different pens. Matthias deliberates what pen to choose. Only after careful pondering he decides and takes one pen.
Deliberation	Picking	Yes	Matthias looks for a new long-term job. He has gotten two job offers. For both jobs he received contracts for signing. The job conditions are identical. Nevertheless Matthias deliberates what job would be better. Only after careful pondering he decides for a job and signs one contract.
Deliberation	Picking	No	Matthias looks for a pen to take a rather unimportant note. On the desk in front of him he sees two identical pens. Matthias deliberates what pen to choose. Only after careful pondering he decides and takes one pen.
Spontaneity	Choosing	Yes	Matthias looks for a new long-term job. He has gotten two job offers. For both jobs he received contracts for signing. The job conditions are very different. Matthias does not deliberate what job to choose. He signs one contract spontaneously.
Spontaneity	Choosing	No	Matthias looks for a pen to take a rather unimportant note. On the desk in front of him he sees two very different pens. Matthias does not deliberate what pen to choose. He takes one pen spontaneously.
Spontaneity	Picking	Yes	Matthias looks for a new long-term job. He has gotten two job offers. For both jobs he received contracts for signing. The job conditions are identical. Matthias does not deliberate what job to choose. He signs one contract spontaneously.
Spontaneity	Picking	No	Matthias looks for a pen to take a rather unimportant note. On the desk in front of him he sees two identical pens. Matthias does not deliberate what pen to choose. He takes one pen spontaneously.


At the beginning, subjects were presented with instructions for completing the questionnaire. We asked the respondents to assess how free/responsible they considered each of eight displayed actions according to their individual beliefs. For each respondent, the order of the scenarios was randomized. The respondent saw only one scenario at a time. Subjects answered using a rating scale with a range from 0 to 100, where 0 indicates “not free/not responsible” and 100 “free/responsible” (depending on the group, they had been assigned to). Please note that in the philosophical literature, freedom and responsibility are frequently considered dichotomous rather than continuous. Here we opt for the continuous scale because it entails the dichotomous case as one possibility for participants to respond. Below the freedom/responsibility rating an additional question was presented that asked, “How confident are you about the rating?” (confidence rating, CR) and was to be answered on a scale from 0 “not certain” to 100 “certain.” This was done to monitor whether subjects had clear beliefs about the different scenarios. There were no time constraints for responding to the questions.

## Results

### Ratings of Freedom and Responsibility

**FIGURE 1:**
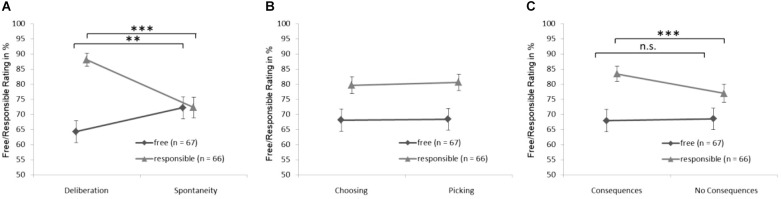
Interaction between rating type separately for each the three within-Subject factors (Collapsed across all other conditions): **(A)** interaction between rating type and deliberation, **(B)** interaction between rating type and choice, and **(C)** interaction between rating type and consequence error bars indicate SEM across all subjects of one group. Asterisks indicates significant difference for *post hoc* analysis (n.s.– *p* > 0.05, ^∗∗^*p* < 0.01, ^∗∗∗^*p* < 0.001).

Figure 1 shows the mean judgements of freedom and responsibility plotted separately for the three main experimental factors (for full results see Table 2). We performed a four-factorial mixed ANOVA with three within-subject experimental factors (Deliberation × Choice × Consequences) and one between-subject factor (Rating Type).

(1) Overall, participants rated the responsibility for actions higher than their freedom, as indicated by a significant main effect of the between-subject factor Rating Type [Figure 1; *F*_(1,131)_ = 15.37, *p* < 0.001, Cohen’s *d* = 0.40].
(2) There was a significant interaction effect between the factors Deliberation and Rating Type [Figure 1A; *F*_(1,131)_ = 35.66, *p* < 0.001, Cohen’s *d* = 1.12]. This strong effect indicates that the factor Deliberation had different effects on ratings of freedom. Deliberating about an action (as opposed to acting spontaneously) led subjects to judge that action as more responsible but less free. The difference between freedom ratings of deliberate versus spontaneous actions was significant *t*(266) = -2.92, *p* = 0.004, Cohen’s *d* = 0.26. The difference between responsibility ratings of deliberate versus spontaneous actions was also significant *t*(288) = 9.07, *p* < 0.001, Cohen’s *d* = 0.56.
(3) There was no significant interaction effect of Rating Type and Choice [Figure 1B; *F*_(1,131)_ = 0.257, *p* = 0.663], indicating that the judgment of freedom versus responsibility was not differentially affected by whether a choice involved different or equal options.
(4) There was a significant interaction between Rating Type and Consequences [Figure 1C; *F*_(1,131)_ = 5.55, *p* = 0.020, Cohen’s *d* = 0.21]. While an action with consequences for a person’s life (compared to an action without consequence) was judged to make a person more responsible *t*(288) = 3.52, *p* > 0.001, Cohen’s *d* = 0.23 it had no effect on the degree to which the action was rated as free *t*(266) = -0.21, *p* = 0.83.

**Table 2:** Descriptive statistics.

Design factors				Rating		Confidence rating (CR)	
Deliberation	Choice	Consequence	Rating type	*M*	*SD*	*M*	*SD*
Deliberation	Choosing	Yes	Freedom	67.07	28.21	75.69	23.81
Deliberation	Choosing	No	Freedom	56.68	32.21	77.81	22.01
Deliberation	Picking	Yes	Freedom	65.12	32.56	71.61	28.98
Deliberation	Picking	No	Freedom	68.19	29.39	74.49	26.20
Spontaneity	Choosing	Yes	Freedom	73.64	28.65	77.53	26.44
Spontaneity	Choosing	No	Freedom	76.76	30.83	67.73	29.99
Spontaneity	Picking	Yes	Freedom	66.47	34.51	78.59	26.78
Spontaneity	Picking	No	Freedom	71.83	27.09	71.24	25.69
Deliberation	Choosing	Yes	Responsibility	86.05	19.51	84.89	19.65
Deliberation	Choosing	No	Responsibility	83.34	25.83	86.86	20.91
Deliberation	Picking	Yes	Responsibility	90.82	16.51	83.80	23.15
Deliberation	Picking	No	Responsibility	91.85	13.14	85.09	21.83
Spontaneity	Choosing	Yes	Responsibility	68.06	28.51	77.35	24.75
Spontaneity	Choosing	No	Responsibility	70.22	29.99	81.39	25.25
Spontaneity	Picking	Yes	Responsibility	73.75	32.14	81.56	24.67
Spontaneity	Picking	No	Responsibility	76.99	25.25	80.26	20.92


### Ratings of Confidence

Throughout the conditions, confidence ratings were high (*M* = 78.50; *SD* = 24.44), ranging from 67.73 to 86.86 (Table 2). Thus, we found no evidence that participants were uncertain how to judge the scenarios.

## Discussion

Our results reveal important dissociations between judgements of freedom and responsibility regarding actions. Overall ratings of responsibility were higher than those of freedom. However, given the between-participant design of the study this overall difference might be a matter of scaling and is thus hard to interpret. The key finding is that the experimental variables affect these two types of ratings differentially: When an action was based on deliberation (rather than being spontaneous), the action was judged to be less free, but its agent was considered to be more responsible for it. When an action involved real-world consequences (vs. not), its agent was considered more responsible, but consequences did not affect the freedom. Whether an action was between equal options or not had no discernible effect on freedom or responsibility ratings.

Our study did not explicitly seek a representative sample (similar to many previous studies, e.g., those using Mechanical Turk). It consisted of a spontaneous sample of respondents responding to an invitation to participate. Overall, the distribution of ages in our sample is not that different than in standard experiments in psychology (mean age 25.03 years, standard deviation 7.76 years; please note that sampling from a Gaussian will always involve a few values from the tails). Our study was thus not designed to resolve the effects of age ranges. In order to address this important point we are currently obtaining data from representative samples on related scenarios, which is the only way to properly address these effects.

Previous studies have used similar designs to assess influences of experimental factors on free will and moral responsibility judgements (Nahmias et al., 2005, 2007). In one study (Nahmias et al., 2005) switching from a negative action (robbing a bank) to a positive action (saving a child) increased moral responsibility ratings but decreased freedom ratings. However, this specific aspect of the study can only be observed descriptively because no direct test for an interaction between these two factors was provided (the focus of the study was otherwise). Another study (Nahmias et al., 2007) showed for different manipulations (switching between neural and psychological determinism, switching between the real world and an alternate world, and switching between good and bad actions) that free will and moral responsibility were generally affected in a similar direction. In contrast, we find that factors such as deliberation and the presence or absence of consequences do have differential effects on free will and responsibility. Based on our previous work (Deutschländer et al., 2017) one could speculate that these experimental manipulations might have been stronger in bringing out the dissociations between free will and responsibility.

In the present data, the freedom ratings considered alone were only affected by deliberation, but not by the nature of the choice (choosing/picking) or by the possible consequences. This is largely in line with a previous study where we found the factor of deliberation to have a moderate effect (Deutschländer et al., 2017), whereas the factors of choice and consequences had only marginal effects. Presumably the minor differences are due to the lower number of participants in the current study.

One question is whether participants could have understood the deliberation vignettes differently. For example, if an agent acted spontaneously, participants might have thought that the agent had reasons but was not aware of them. In that case the difference between acting spontaneously versus deliberately was that the agent was aware of their reasons if they acted deliberately while they were not aware of their reasons if they acted spontaneously. In order to investigate this alternative interpretation, future research should distinguish between having reasons, being aware of those reasons, and forming reasons by deliberation. Furthermore, future studies could provide a more in-depth assessment between judgements of free will and responsibility by directly probing individual participants on both concepts within a single study.

Another interesting question is whether the freedom or responsibility effects pertain to the agent’s *action* or to the *situation*. In our first main finding the experimental manipulation is independent of the situational context: The difference between deliberative and spontaneous is only in the internal mental process, while the external conditions remain exactly the same. Here, the key effect of deliberation versus spontaneity can thus not be explained by differences in external conditions. In contrast, our second main finding of an effect of consequence involves a change in the situation the agent is in. However, please note that also in this scenario the participants were asked to rate the freedom / responsibility of the action, not the situation.

Another question is how exactly participants understood the factor Consequence. Participants have rated an agent as more responsible for an action with consequences than without consequences. When there are consequences of an action, there is more for the agent to be responsible for, so the agent is responsible for more. However, this is not to say that he has more responsibility. I can kill and steal with equal responsibility, even if I am responsible for more in the killing case. Participants might mistake the degree of responsibility of an agent with the harms the action causes. A potential follow-up needs to distinguish degrees of responsibility from degrees of harms for which a person is responsible in order to clarify what the participants had in mind.

Another interesting implication of our findings relates to Libet-style experiments (Libet et al., 1983) Some researchers interpret the results of Libet’s experiments as evidence that human freedom is illusory and therefore the concept of responsibility also needs to be revised (Wegner, 2002). Besides criticism by empirical researchers (Schurger et al., 2012; Schultze-Kraft et al., 2016), especially Philosophers have pointed out a number of serious objections against the Libet-style experiments and their radical interpretation (Sinnott-Armstrong and Nadel, 2011). Among those objections, one particular critique seems to be affected by our results. Some philosophers have suggested that the actions in Libet-style experiments do not qualify as free, because they lack reasons, distinguishable options, and real life consequences. “Arbitrary action (i.e., Libet Action) is at best a degenerate case of freedom of will, one in which what matters fails to hold” (Roskies, 2011, p. 18). Our results suggest that this particular objection might fail for the folk concept of *freedom* but still succeed for the folk concept of *responsibility*. From a folk perspective, actions in Libet-style experiments qualify as free action even if they are spontaneous, without much of a choice, and without consequences. The dissociation between freedom and responsibility in our study thus means that the Libet-style experiments do not speak to the issue of responsibility.

In general, the differential effects of deliberation on freedom and responsibility ratings raise questions as to whether freedom is considered a necessary condition for responsibility by lay people. We do not consider our folk psychological finding to mean that philosophers should avoid postulating this necessity, but our results serve as a warning that this necessity might not be intuitive, which is an important consideration given the immense public interest and engagement in the free will debate (e.g., Overbye, 2007). Please note, that many philosophers have argued that their positions should be in line with lay beliefs (Jackson, 2000).

Our study might help to restructure debates about freedom and responsibility and partially alleviate the tension between neuroscience and psychology, which sometimes claim to refer to lay definitions of these terms (Libet et al., 1983; Libet, 1985, 2005), and philosophy, which often employs more elaborate definitions of freedom and responsibility (Roskies, 2011). Lay intuitions of *freedom* are not in line with some common philosophical theories, because lay people ascribe more freedom in conditions of spontaneity and in the absence of reasons (Deutschländer et al., 2017). However, the lay intuitions regarding *responsibility* are very well aligned with claims by many philosophers that responsibility requires consideration of reasons. These compatibilist philosophers do not normally think that actual deliberation is crucial for responsibility but only that the agent has to be able at least in principle to respond to reasons, an ability typically coined reason-responsiveness (Fischer and Ravizza, 1998).

This account of responsibility opens up the possibility that agents sometimes have responsibility without freedom and that determinism is compatible with responsibility but not with freedom. Some philosophers (Fischer, 2006) and scientists (Gazzaniga, 2012) have explicitly endorsed this position, which is called semi-compatibilism. Our findings follow a pattern that would be expected if laypeople were to hold semi-compatibilist beliefs, according to which the ability to adequately consider reasons in deliberation increases responsibility but is not necessary for and might even reduce the sense of freedom. An interesting question is whether our results also extend to actions that are explicitly irresponsible (as opposed to less responsible). Our results don’t speak to this clearly enough because overall our responsibility ratings were high. However, this is certainly an interesting question for future research.

Our experiments obviously cannot prove directly that lay people are semi-compatibilists, as we did not ask them explicitly about their views on the relationship between determinism and freedom or moral responsibility. We doubt that lay people have stable, developed, or detailed views about such abstract theoretical notions^1^. Nonetheless, our studies do show that notions like *reason* and *deliberation,* which form an integral part of the necessary abilities for responsible agency according to semi-compatibilists, are in fact also positively associated with responsibility in the mind of lay people, in contrast with lay intuitions on freedom. The gap between the intuitions of lay people, scientific results and philosophical theorizing in this respect might be less deep than often assumed.

## Ethics Statement

Study approved by the Ethics Committee of the Institute for Psychology, Humboldt University Berlin.

## Author Contributions

RD designed the study and analyzed the data. TV designed the study. WS-A conceptualized the input. J-DH designed the study and supervised the data analysis. All authors wrote the manuscript.

## Conflict of Interest Statement

The authors declare that the research was conducted in the absence of any commercial or financial relationships that could be construed as a potential conflict of interest.

## Footnotes

1 The debate over lay intuitions on determinism and compatibilism between Nahmias (Nahmias et al., 2005, 2007) and Nichols and Knobe (2007) seems very much to support this point.

## References

[B1] Aristotle (2000). *Nicomachean Ethics.* Oxford: Oxford University Press.

[B2] Augustine (2006). *De libero arbitrio = : Der freie Wille.* Paderborn: Ferdinand Schöningh.

[B3] Deutschländer R. Pauen M. Haynes J.-D. (2017). Probing folk-psychology: do Libet-style experiments reflect folk intuitions about free action? *Conscious. Cogn.* 48 232–245. 10.1016/j.concog.2016.11.004 28013177

[B4] Fischer J. M. (2006). *My way: Essays on Moral Responsibility.* Oxford: Oxford University Press.

[B5] Fischer J. M. Ravizza M. (1998). *Responsibility and Control a Theory of Moral Responsibility.* Cambridge: Cambridge University Press.

[B6] Gazzaniga M. (2012). *Who’s in Charge?: Free Will and the Science of the Brain.* London: Hachette.

[B7] Jackson F. (2000). *From Metaphysics to Ethics.* Oxford: Oxford University Press.

[B8] Kane R. (2005). *A Contemporary Introduction to Free Will.* Oxford: Oxford University Press.

[B9] Kant I. (1998). *Critique of Pure Reason.* Cambridge: Cambridge University Press.

[B10] Libet B. (1985). Unconscious cerebral initiative and the role of conscious will in voluntary action. *Behav. Brain Sci.* 8 529–539.

[B11] Libet B. (2005). *Mind Time: The Temporal Factor in Consciousness.* Boston, MA: Harvard University Press.

[B12] Libet B. Gleason C. A. Wright E. W. Pearl D. K. (1983). Time of conscious intention to act in relation to onset of cerebral activity (Readiness Potential). *Brain* 106 623–642. 10.1093/brain/106.3.623 6640273

[B13] Locke J. (1975). *The Clarendon Edition of the Works of John Locke: An Essay Concerning Human Understanding.* Oxford: Oxford University Press.

[B14] Mecacci G. Haselager P. (2015). A reason to be free. *Neuroethics* 8 327–334. 10.1007/s12152-015-9241-8

[B15] Nahmias E. Coates D. J. Kvaran T. (2007). Free will, moral responsibility, and mechanism: experiments on folk intuitions. *Midwest Stud. Philos.* 31 214–242. 10.1111/j.1475-4975.2007.00158.x

[B16] Nahmias E. Morris S. Nadelhoffer T. Turner L. J. (2005). Surveying freedom: folk intuitions about free will and moral responsibility. *Philos. Psychol.* 18 561–584. 10.1080/09515080500264180

[B17] Nichols S. Knobe J. (2007). Moral responsibility and determinism: the cognitive science of folk intuitions. *Nous* 41 663–685. 10.1111/j.1468-0068.2007.00666.x

[B18] Overbye D. (2007). *Free Will: Now You Have It, Now You Don’t. The New York Times*. Available at: http://www.nytimes.com/2007/01/02/science/02free.html#addenda (accessed May 7 2019).

[B19] Roskies A. L. (2011). “Why Libet’s studies don’t pose a threat,” in *Conscious Will and Responsibility*, eds Sinnott-Armstrong W. Nadel L. (Oxford: Oxford University Press), 11–22. 10.1093/acprof:oso/9780195381641.003.0003

[B20] Schlosser M. E. (2014). The neuroscientific study of free will: a diagnosis of the controversy. *Synthese* 191 245–262. 10.1007/s11229-013-0312-2

[B21] Schultze-Kraft M. Birman D. Rusconi M. Allefeld C. Görgen K. Dähne S. (2016). The point of no return in vetoing self-initiated movements. *Proc. Natl. Acad. Sci. U.S.A.* 113 1080–1085. 10.1073/pnas.1513569112 26668390 PMC4743787

[B22] Schurger A. Sitt J. D. Dehaene S. (2012). An accumulator model for spontaneous neural activity prior to self-initiated movement. *Proc. Natl. Acad. Sci. U.S.A.* 109 2904–2913. 10.1073/pnas.1210467109 PMC3479453 22869750

[B23] Sinnott-Armstrong W. Nadel L. (eds) (2011). *Conscious Will and Responsibility.* Oxford: Oxford University Press.

[B24] Ullmann-Margalit E. Morgenbesser S. (1977). Picking and choosing. *Soc. Res.* 44 757–785.

[B25] Van Inwagen P. (1983). *An Essay on Free Will.* Oxford: Clarendon Press.

[B26] Van Inwagen P. (1989). When is the will free? *Philos. Perspect.* 3 399–422.

[B27] Vihvelin K. (2008). *Arguments for Incompatibilism. in Stanford Encyclopedia of Philosophy.*Available at: https://plato.stanford.edu/entries/incompatibilism-arguments/ (accessed May 7 2019).

[B28] Wegner D. (2002). *The Illusion of Conscious Will.* Cambridge, MA: MIT Press.

